# Evidence-based surgical technique for medial unicompartmental knee arthroplasty

**DOI:** 10.1186/s43019-020-00084-x

**Published:** 2021-01-07

**Authors:** Tae Kyun Kim, Anurag Mittal, Prashant Meshram, Woo Hyun Kim, Sang Min Choi

**Affiliations:** 1 TK Orthopedic Institution, 55 Dongpangyo-ro, Bundang-gu, Seongnam-si, Gyeonggi-do 13535 Republic of Korea; 2 Department of Orthopaedics, Johns Hopkins Medical Institute, 2360 West Joppa Road, Suite 306, Baltimore, MD 21093 USA

**Keywords:** Unicompartmental knee arthroplasty, Knee replacement, UKA surgical technique, Persona partial knee

## Abstract

Unicompartmental knee arthroplasty (UKA) is a successful treatment modality in selected patients having advanced, single-compartment osteoarthritis of the knee. The bone and ligament preservation leading to shorter recovery periods, better functional outcomes, lower perioperative complication rates, and easier revision, if needed, are proposed as some of the advantages of UKA over total knee arthroplasty (TKA). Despite several advantages, UKA is reported to have higher failure rates as compared to TKA. The prosthesis failure of UKA is directly correlated to intraoperative technique-related factors like malpositioning of components and the inability to replicate the target-limb alignment as per preoperative planning. An evidence-based surgical technique for UKA may help surgeons to avoid the intraoperative technique-related errors. The purpose of this paper is to describe a stepwise surgical technique for the fixed-bearing medial UKA.

## Introduction

The surgical options for patients with advanced osteoarthritis of a single compartment of the knee are unicompartmental knee arthroplasty (UKA), high tibial osteotomy (HTO), and total knee arthroplasty (TKA) [1]. UKA surgery is regarded as a less aggressive surgery than TKA, by virtue of the ligament preservation and reduced bone resection that it entails. As compared with TKA, UKA has been shown to have a shorter operative time, less blood loss, shorter hospital stays, earlier rehabilitation, improved gait kinematics, better range of motion, and the possibility of earlier return to sports [2–6]. In comparison to HTO, UKA offers a safe and efficient alternative with reduced postoperative pain, fewer postoperative complication, and revision especially in the older and less active population [7]. Notably, a 10-year survivorship of 93% has been reported in a cohort of Korean patients undergoing UKA [8]. The contemporary emphasis to improve survivorship and function after UKA is on optimizing patient selection and improving surgical technique to reduce surgical errors.

In recent years, the number of UKAs performed has remained steady or shown rising trends globally [9–12]. One of the reasons for the increase in the number of UKAs performed globally is its expanding indications [13]. More recent inclusion criteria for medial UKA include advanced medial compartment osteoarthritis or osteonecrosis, functional anterior cruciate ligament (ACL), preservation of the lateral compartment, passively correctible varus deformity of less than 10°, fixed-flexion deformity less than 15°, maximum knee flexion greater than 90°, and patient age of between 18 and 80 years [14–23]. Exclusion criteria include a diagnosis of inflammatory arthritis, hemochromatosis, hemophilia, symptomatic knee instability, multi-compartment disease, previously failed correctional osteotomy or ipsilateral UKA, immobility, or any other neurological condition affecting musculoskeletal function. Nonetheless, young age, high level of activity, obesity, presence of lateral osteophytes, chondrocalcinosis, and low-grade patellofemoral arthritis are not considered as absolute contraindications of UKA, as previously thought [9, 20].

Besides careful patient selection, another way to improve survivorship of UKA is by improving the surgical technique to avoid errors. The technical difficulties relating to limited surgical exposure and narrow margin of error in achieving surgical goals are one of the major reasons for surgeons hesitating to choose UKA over TKA. About 50% of the failures after UKA occur within the first 5 years of surgery [24, 25]. These are attributed to surgical-technique-dependent failures which include aseptic loosening, polyethylene liner dislocation, and disease progression to another compartment [26–30]. While performing UKA surgery, the malpositioning of components and the inability to replicate the target-limb alignment as per preoperative planning have been shown to correlate with worse prosthesis survival and clinical outcomes [31–35]. While computer-assisted technologies, like navigation and robotics, are proposed to minimize the surgical errors, the evidence on their clinical efficacy is limited and there are concerns of low cost-effectiveness [36, 37].

Apart from the accuracy of bone resection and implant positioning, there are several key surgeon-controlled factors which are critical to the success of UKA that can be performed without computer assistance. These steps include, but are not limited to, the length of incision, approach and extent of exposure, cementation, and soft-tissue handling [38]. Thus, it becomes necessary for surgeons to adopt an evidence-based and meticulous surgical technique to optimize the results of UKA. Moreover, there is a learning curve ranging from 16 to 29 cases associated with conventional UKA that must be considered by novel surgeons [39, 40]. As the difficulties in adopting a new procedure have been highlighted in the past, an account of a stepwise approach to perform UKA may help surgeons to adopt this surgery in their practice [41]. To the best of our knowledge, there are no scientific articles in the literature describing an evidence-based surgical technique of UKA with a contemporary fixed-bearing UKA design. The purpose of this paper is to describe a stepwise surgical technique based on the available scientific evidence for the medial UKA using the *Persona® Partial Knee* (PPK, Zimmer, Warsaw, IN, USA) system.

## Surgical technique

We perform fixed bearing medial UKA (PPK) in patients having bone on bone arthritis of medial compartment of knee with normal lateral compartment and passively correctible deformities as described above. Launched in 2017, PPK features an anatomical tibial tray for maximal bone coverage without overhang, a conforming femur design and a fixed-bearing polyethylene. The surgical procedure is performed using a spacer block technique. The surgical technique for UKA using a PPK prosthesis as routinely performed by the senior author (TKK) has been discussed in the following nine steps with tips and pearls. The rationale of performing every step based on the scientific literature, when available, or anecdotal experience is also provided. The nine steps enlisted below are demonstrated in video1.

1. Exposure: (a) skin incision, (b) arthrotomy (c) dissection
2. Proximal tibial resection: (a) vertical-cut marking, (b) extramedullary (EM) jig placement and vertical cut, (c) horizontal cut
3. Distal femoral resection: (a) spacer block method, (b) gap check
4. Femoral sizing and final femoral preparation: (a) posterior rasping, (b) femoral size determination and finishing guide placement, (c) final preparation, (d) femoral trial test
5. Gap assessment
6. Tibial sizing and final tibial preparation
7. Trial test and polyethylene insert selection
8. Implant cementation: (a) tibial cementation, (b) femoral cementation, (c) final assessment and implantation
9. Placement of polyethylene insert and wound closure

### Exposure

Adequate surgical exposure is critical in UKA for the visualization of bony and soft-tissue landmarks to avoid suboptimal positioning of the tibial and femoral components. Good exposure also avoids inadvertent tissue damage by forceful retraction. These factors, if not avoided, could indirectly result in inferior functional outcome and decreased survivorship of implants [42, 43].

#### Skin incision

**Fig. 1:**
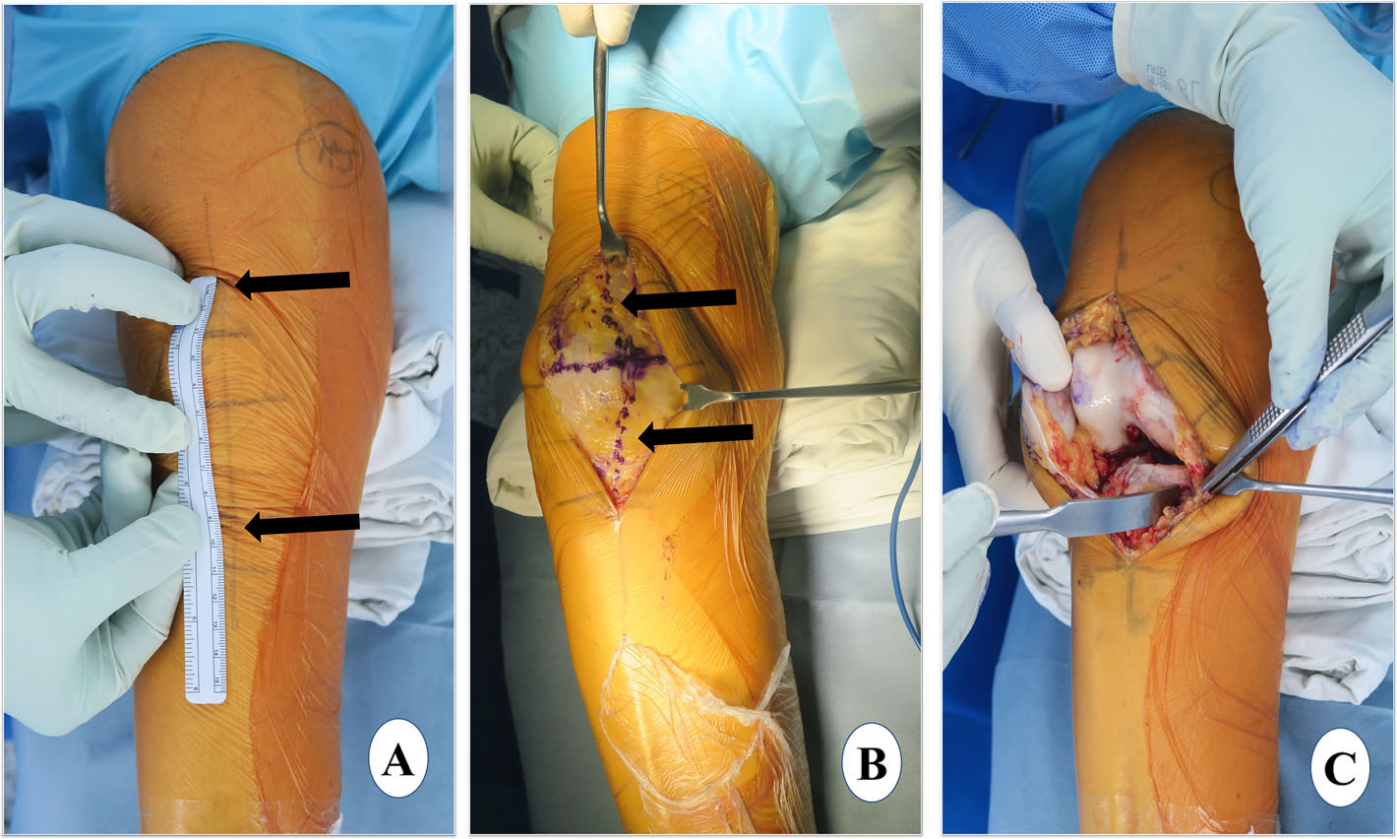
Exposure. **a** skin marking. Arrows show the extent of incision from the superior pole of the patella to a point medial to the tibial tubercle. Ruler shows the length of the incision, **b** arthrotomy marking, and **c** medial envelope development

The patient is laid supine with the knee in 30° of flexion and skin marking is done (Fig. 1a). Alternate long and short transverse markings should be made on the vertical skin marking at a distance of around 2 cm which will be helpful in the closure of the skin at the end of surgery and avoid dog ears. After skin markings, an incision just medial to the midline is made extending from the superior pole of the patella to a point medial to the tibial tubercle *(approximately 10–12 cm)*. In contrast to a more medial incision, the above-mentioned incision will facilitate exposure for a subsequent revision to TKA, if necessary. Also, the tibial tuberosity can be seen easily and used as a landmark for tibial component rotation with a midline incision. The incision should be optimal (not minimal), long enough for easy visualization of the cruciate ligaments, medial tibial plateau, lateral compartment, patellofemoral articulation, vertical line for the tibial cut, and placement of a bent Hohman retractor, Z retractor and extramedullary guide. The incision may have to be extended according to the amount of fat, build of the patient, and muscle tone and deformity in the knee.

#### Arthrotomy

The medial subcutaneous tissue is dissected, and the medial skin flap is retracted with superiorly and medially placed retractors. The arthrotomy line is marked, and it will be helpful to make a horizontal mark in the middle of the patella which will later allow for an accurate closure of the joint capsule. A medial parapatellar arthrotomy is performed extending from the superior pole of the patella curving down distally around the patella and patellar tendon (Fig. 1b). It is advisable to leave 1 cm of tendon attached to the patella for easy capsule closure.

#### Dissection

The lateral prepatellar fat pad is removed for better exposure of the cruciate ligaments and lateral compartment. The anterior one third of the medial meniscus is excised at the menisco-synovial junction using a sharp knife. Then, with slow and continued external rotation of the tibia, a medial envelope comprising the superficial medial collateral ligament (MCL) and capsule is created with a 1 cm-wide, curved osteotome (Fig. 1c). The same osteotome is placed in the posteromedial corner of the proximal tibia. The deep MCL release is completed with electrocautery. Although controversial, we believe that deep MCL release similar to that in TKA should be considered in UKA also because it allows the placement of a Z retractor protecting the superficial MCL [44, 45] This also provides a good exposure of the medial tibial plateau for easy instrumentation and cementation. With the knee in extension and the patella everted, patelloplasty is performed which involves the demarcation of the patellar rim with the help of electrocautery and removal of osteophytes.

The knee is then flexed to 120°. The patella is retracted, and the osteophytes from the medial femoral condyle are removed. The femoral attachment of the MCL is retracted, and the osteophytes underneath are removed with the help of a 1-cm, straight osteotome and a *rongeur*. The osteophytes from both margins of the intercondylar notch and around the anterior cruciate ligament (ACL) are removed with the same instruments. All the osteophytes should be removed before balancing the flexion and extension space. Osteophyte removal will reveal the true deformity of the knee and accurate sizing of the femoral component will be possible. After osteophyte removal, at the time of exposure, the joint should be examined and inspected for the following: the ACL should be functionally intact, there should be bone-on-bone osteoarthritis on the medial compartment, a low grade of patellofemoral arthritis is acceptable, the lateral compartment cartilage should be full thickness, and minimal lateral osteophytes can be tolerated.

### Proximal tibial resection

While performing UKA, the proximal tibial cut is one of the most critical steps, which, if not performed precisely, increases the risk of tibial prosthesis malalignment. This is even more important while using the extramedullary guide and spacer block technique compared to an intramedullary (IM) guided technique due to the possibility of transferring a tibial malalignment to a femoral malalignment [46].

#### Vertical-cut marking

**Fig. 2:**
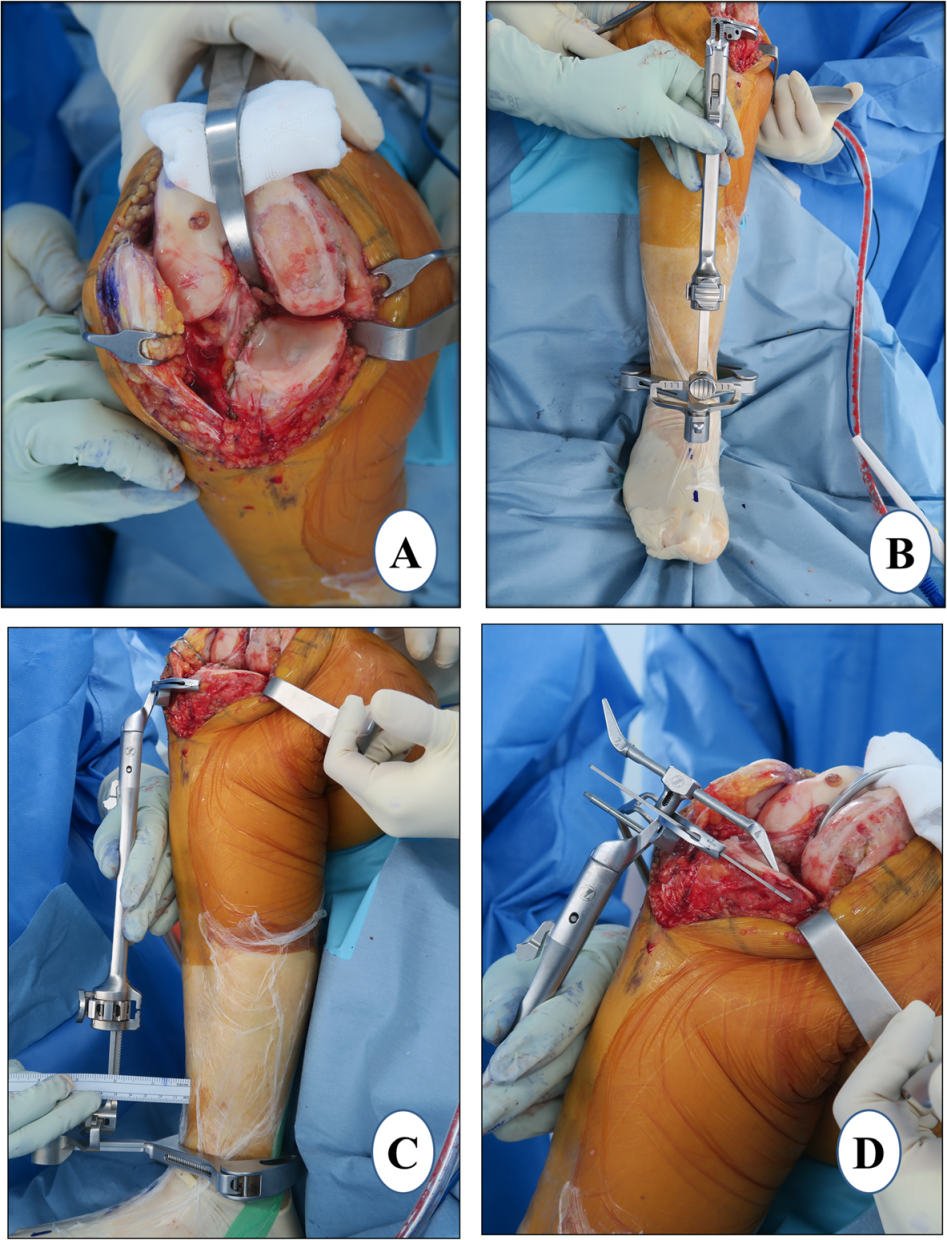
Proximal tibial resection. **a** vertical-cut marking, **b** coronal alignment, **c** tibial slope setting, and **d** resection depth measurement

For the lateral extent (sagittal) of the tibial cut, marking is done with the help of electrocautery. The cut starts posteriorly just medial to the posterior cruciate ligament (PCL) attachment. *It extends anteriorly, violating up to the medial quarter of the fibers of the ACL which do not affect the function of the ACL. Further, the cut runs anteriorly to the medial tibial spine and finally to the medial one third of the tibial tuberosity* (Fig. 2a). This marking sets the tibial component rotation. A blunt-tip reciprocating blade is preferred to make this cut in order to avoid damaging the posterior neurovascular structures. While leaving a flake of cortical bone posteriorly, the vertical cut should run in the anterior to posterior direction. The notch osteophyte removal done previously ensures that the blade of the reciprocating saw is parallel to the femoral condyle and as close to the tibial spine as possible. The cut should be as lateral as possible without violating the integrity of the ACL to the extent that it may result in functional laxity. This helps in accommodating the larger size of the tibial component and thereby avoids undersizing. It also leads to better central loading along with enough support for the tibial tray. These two factors will help to prevent the eccentric loading and subsequent tibial-plateau fracture or medial-wall collapse.

#### Extramedullary (EM) jig placement and vertical cut

With the knee in 120° of flexion and the tibia externally rotated, the anterior and middle tibial osteophytes are removed. With minimal patellar retraction, the tibia is levered out anteriorly with the help of a curved Hohmann retractor. It is important to put the retractor tip over a folded gauze placed posteriorly in the intercondylar notch just medial to PCL attachment to protect the posterior neurovascular structures. A Z retractor is applied over the medial tibial side to protect the posteromedial capsule and superficial MCL. Using a flexible ruler, the mid-point of the line joining the most prominent point of the medial and lateral malleoli is marked. A point 1 cm medial to the midpoint of the tibial surface is taken and extended distally to the first metatarsal and second toe, and proximally in line with the tibial shin. The EM jig is placed along this line which will help to adjust the coronal alignment of the tibial component (Fig. 2b). The slope of the tibial cut is chosen by adjusting the relative distance of the EM rod from the proximal and distal reference points, according to the preoperative planning (Fig. 2c). It is checked with the angel wing to make sure that the anterior and posterior medial tibial thickness being resected is equal and the cutting block is parallel to the medial tibial-plateau slope. In UKA, we generally cut along the natural slope (7–9°). Sometimes, the tibial-cut guide has a built-in posterior slope which a surgeon should be aware of (the PPK tibial-cut guide has 5° of posterior slope built in). A central pin is placed in the EM jig to control the rotation of the cutting block with the coronal and sagittal alignment considered. The two pins are then placed connecting the proximal tibial cutting block to the tibia when all the parameters and resection depth are satisfactory. Subsequently, the vertical cut is made along the marked line described above. *A deeper vertical cut than the intended horizontal cut should be avoided to prevent creating a stress riser. In order to prevent a deeper than intended vertical cut, the lateral corner pin inserted into the sagittal cut slot on the tibial resection guide should be used a marker*.

#### Horizontal cut

This cut will determine the depth of the tibial resection, coronal alignment, and the posterior slope. Generally, the thickness of the tibial cut is 4 mm which is achieved by placing a 4-mm stylus over the most damaged part on the medial tibial condyle and subsequently confirmed with the angel wing (Fig. 2d). We believe that a cut thicker than 4 mm exposes the cancellous metaphyseal bone which may lead to inferior bone prosthesis fixation and risk of early tibial subsidence and medial collapse. This depth is also appropriate for maintaining the native joint line and creating a pre-osteoarthritis alignment, a critical goal of UKA. While checking the gap balance, if the surgeon feels that the knee joint is too tight, then the tibial cut could be revised. Of note, a thinner cut may occasionally be desired if there is severe wear of the tibia. In this case, the 2-mm tip of the stylus can be used. Two pins used for temporarily fixing the tibia-cutting jig may be left in place which will facilitate the tibial cut revision, if necessary. With the Z retractor placed over the medial aspect of the tibia, the horizontal cut is made with a 1-cm oscillating saw through a cutting block slot. It should be ensured that the retractor lies between the saw and the MCL, protecting the deep fibers of the ligament. A lateral straight retractor, a thin, metal device, can be inserted into the notch made by the vertical cut to prevent the saw blade from undermining the tibial eminence laterally.

At this stage, the EM tibial jig is detached. The resected part of tibial-plateau bone is removed with the help of a flat, wide osteotome. The knee may be flexed to 10–15° to assist with the removal of the resected tibial plateau. The excised tibial plateau should be examined for the features of isolated anteromedial arthritis such as full-thickness cartilage loss anteriorly and relative preservation of the cartilage posteriorly. If this pattern is not observed, the status of the ACL is re-assessed. The anterior and posterior thickness of the excised tibial plateau is measured; it should be equal and preferably be 4 mm, indicating an optimal posterior slope. A spacer block preferably of 9 mm is inserted with an alignment guide to check the requisite coronal alignment, tibial slope, and the tightness in 5–10° of flexion. It is recommended to use a 9-mm spacer block instead of an 8-mm spacer block as a 9-mm spacer allows the surgeon intraoperative flexibility of ± 1 mm [47]. The thickness is correct when the 9-mm spacer block can slide easily in and out. If the 9-mm spacer block is too loose, use a thicker spacer block to fill the extension gap. If the 9-mm spacer block has to be firmly gripped to slide in and out, this is too tight, and additional tibial resection is required.

#### Evidence

##### Mechanical hip-knee-ankle axis

The evidence suggests that 1–4° of varus alignment should be the goal in UKA [48]. This will prevent overloading the lateral compartment and subsequent lateral compartment osteoarthritis which is one of the common reasons for UKA failure.

##### Tibial coronal alignment

Inappropriate coronal alignment of the tibial component could place undue stress on both cortical and cancellous bone, which may compromise the stability and long-term survivorship of the prosthesis [49]. The clinical results tend to be poor when the tibial components are placed in varus coronal alignment [50]. As far as bone stress and contact pressure on the bone-metal interface are concerned, a previous study has found that a slight valgus is the most stable inclination for the tibial component of UKA [51]. *The coronal alignment of the UKA tibial and femoral components does not affect the FTA, as long as the joint line is maintained* [52].

##### Tibial component rotation

During normal flexion, the tibia rotates internally around 20°. The lateral compartment moves posteriorly more or less double the distance than the medial compartment [53]. The tibial rotation during range of motion is similar in osteoarthritic knees and knees after UKA [53]. Thus, a neutral rotation in extension and a 20° external rotation in flexion indicates the ideal position of the tibial component. Theoretically, a rotation around 10° could be appropriate for optimal knee kinematics neither in flexion nor extension [54]. However, in clinical studies, a tibial component axial rotation within 3° of external rotation to 3° of internal rotation has been correlated with significantly better clinical outcomes and functional scores [54, 55].

##### Tibial slope

The tibial slope is an important factor that controls tibial translation during weight-bearing with an unconstrained implant design such as PPK. The posterior tibial slope is important for knee stability. A slight increase of the posterior tibial slope has often been preferred to promote increased flexion and femoral rollback and to improve the stress distribution at the bone-tibial component interface. A posterior slope of between 3° and 7° is recommended to balance between the risk of excessive translation on one hand with the risk of excessive stress and cruciate ligament avulsion on the other hand [56, 57].

##### Tibial resection depth

If the medial tibial surface is moved distally either by cutting excess bone prior to placement of the tibial component or using a thin polyethylene insert, the mechanical axis of the lower extremity shifts medially [58]. This will increase the mechanical stress on the medial compartment (increase the FTA) and may cause aseptic loosening of the components. Thus, the depth of the tibial cut should be kept to a minimum possible amount.

### Distal femoral resection

This femoral cut will define the coronal alignment of the femur. When the spacer block method is used for femoral preparation, the distal femoral cut is made according to the tibial cut. It should be noted that the thickness of the spacer block does not change the thickness of the femoral cut. The spacer, as the name suggests, only fills the space between the tibia and femur to make the knee stable.

**Fig. 3:**
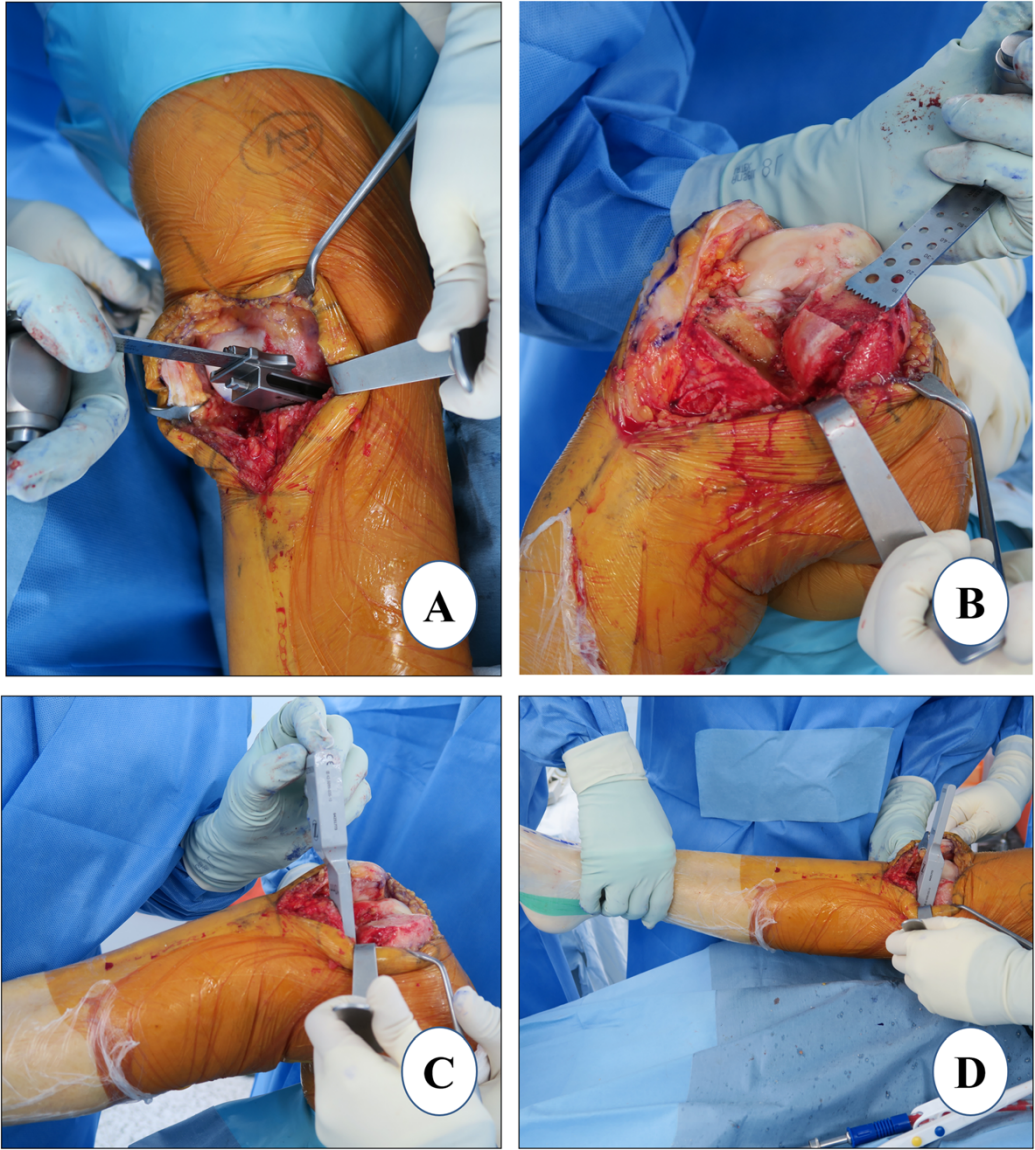
Distal femoral resection. **a** spacer block method, **b** manual completion of distal femoral cut, **c** flexion gap check with thin blade, and **d** extension gap check with thick blade

With the knee in extension, a spacer block of 9 mm or the thickness used earlier to confirm the tibial resection is inserted (Fig. 3a). It is important to confirm that the spacer block is fully inserted and sitting flat on the resected tibia and in contact with the distal femur. Anterior femoral osteophytes, if found, should be removed. Care should be taken not to hyperextend the knee. The narrow blade of the Z retractor is placed underneath the superficial MCL to prevent injury (Fig. 3a). While making the cut, surgeons should be careful not to go laterally as it may injure the trochlear groove. It would be advisable to cut half of the distal femoral surface with the spacer block in situ and the rest of the cut could be completed freehand using a wide, oscillating saw (Fig. 3b). The knee could be placed in mid-flexion while completing the remaining posterior half cut to prevent damage to the posterior neurovascular bundles.

#### Gap check

The flexion gap is measured in about 100° flexion using the thin blade of the gap checking device that matches the spacer block thickness used for cutting the distal femur (Fig. 3c). The extension gap is measured with the thick blade of this device (Fig. 3d). The extension gap should be measured in 5–10° of flexion as posterior structures become tight in extension and may result in underestimation. Confirmation of the correct gap is assessed subjectively by checking whether a thicker gap checker is difficult to insert and a thinner gap checker is loose. It is important to ensure a slight undercorrection of the limb alignment and have at least 2 mm of laxity in extension as well as in flexion.

#### Evidence

An intramedullary guide and spacer block method are two techniques used in UKA to make the distal femoral cut. The spacer block technique aligns the distal resection of the femur parallel to the tibial resection in extension. The intramedullary technique requires perforation of the trochlea for guide placement, which, unlike TKA, is not replaced with a part of prosthesis in UKA. Extramedullary (EM) techniques avoid cannulating the medullary canals of the femur or tibia, diminishing the chances of marrow emboli and bleeding from the canal [59]. In a retrospective study, a 92.2% survival rate at a mean of 5.7 years’ follow-up was observed using EM tibial and femoral guides in 128 UKAs [60]. Both techniques have been shown to result in similar soft-tissue balancing and femoral alignment [61, 62]. Thus, we believe that the spacer block method is a better alternative.

### Femoral sizing and final femoral preparation

#### Femoral sizing

**Fig. 4:**
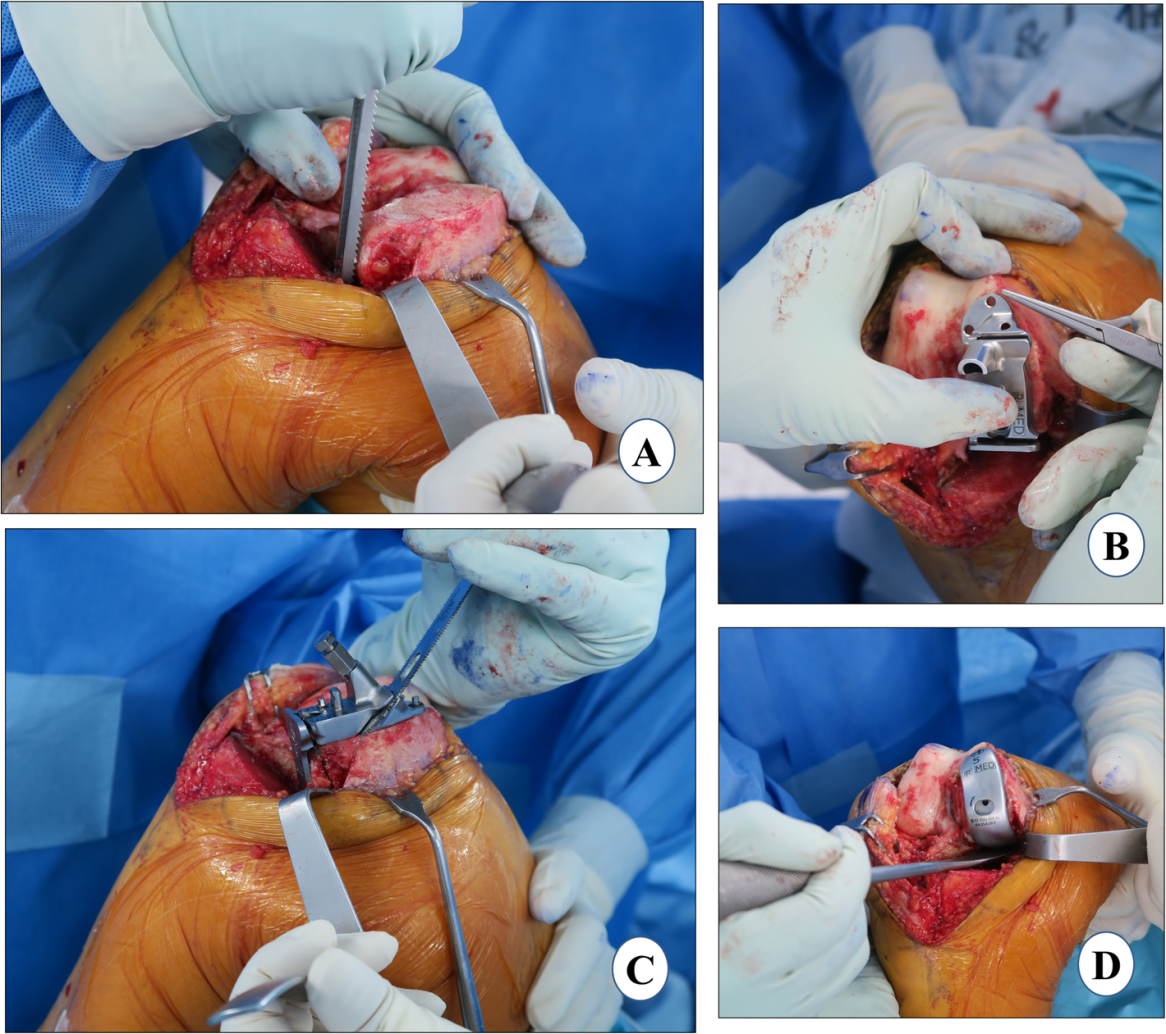
Femoral sizing and final femoral preparation. **a** posterior rasping, **b** femoral finishing guide placement, **c** posterior femoral cut, and chamfer cut, and **d** femur trial

If the flexion gap is tight, any intact cartilage in the posterior femoral condyle should be rasped to anteriorize the femoral component (Fig. 4a). With the knee in ~ 100° of flexion, the femoral final “finishing” guide is placed on the resected distal femoral surface and the retained posterior condyle in order to assess the appropriate femoral size (Fig. 4b). The profile of each femoral finishing guide matches the location and profile of the corresponding femoral component anteriorly and distally. The guide should be lateralized as far as possible, aligning to the lateral border of the medial femoral condyle to avoid impingement on the intercondylar notch. This will increase the likelihood of the tibial component to properly track with the femur in extension and prevent patellofemoral impingement [63]. When properly sized, there should be a rim of at least 2 mm of exposed bone, anterior and medial to the femoral finishing guide (Fig. 4b). When confused between two subsequent femoral component sizes, the surgeon should choose the smaller size to prevent overhang which can lead to patellar impingement. The posterior edge of the guide should be parallel to the proximal tibial cut surface which can be ensured by insertion of the thin side of the flexion/extension gap checker underneath the femoral finishing guide. This is important to prevent femur-tibia prosthesis divergence. Flexion malalignment of the femoral component should be avoided, because it could lead to adverse contact stress on the polyethylene insert and articular cartilage of the lateral compartment [64].

#### Final preparation

Once, the desired position is achieved, the finishing femoral guide is secured with three pins. Anterior peg-hole preparation is performed, and a lug rod is placed to provide support for the cutting block. Then, posterior peg-hole preparation is performed. The posterior femoral and posterior chamfer cuts are executed sequentially using the reciprocating saw with the Z retractor placed to protect the MCL (Fig. 4c). Again, it is advisable to carry out these cuts only partially with the guide, the rest should be done freehandedly. The trial component is fitted to confirm the correct femoral size (Fig. 4d). If the uncovered bone posteriorly is more than 2 mm, the bone is removed using the posterior-curved osteotome.

### Gap assessment

**Fig. 5:**
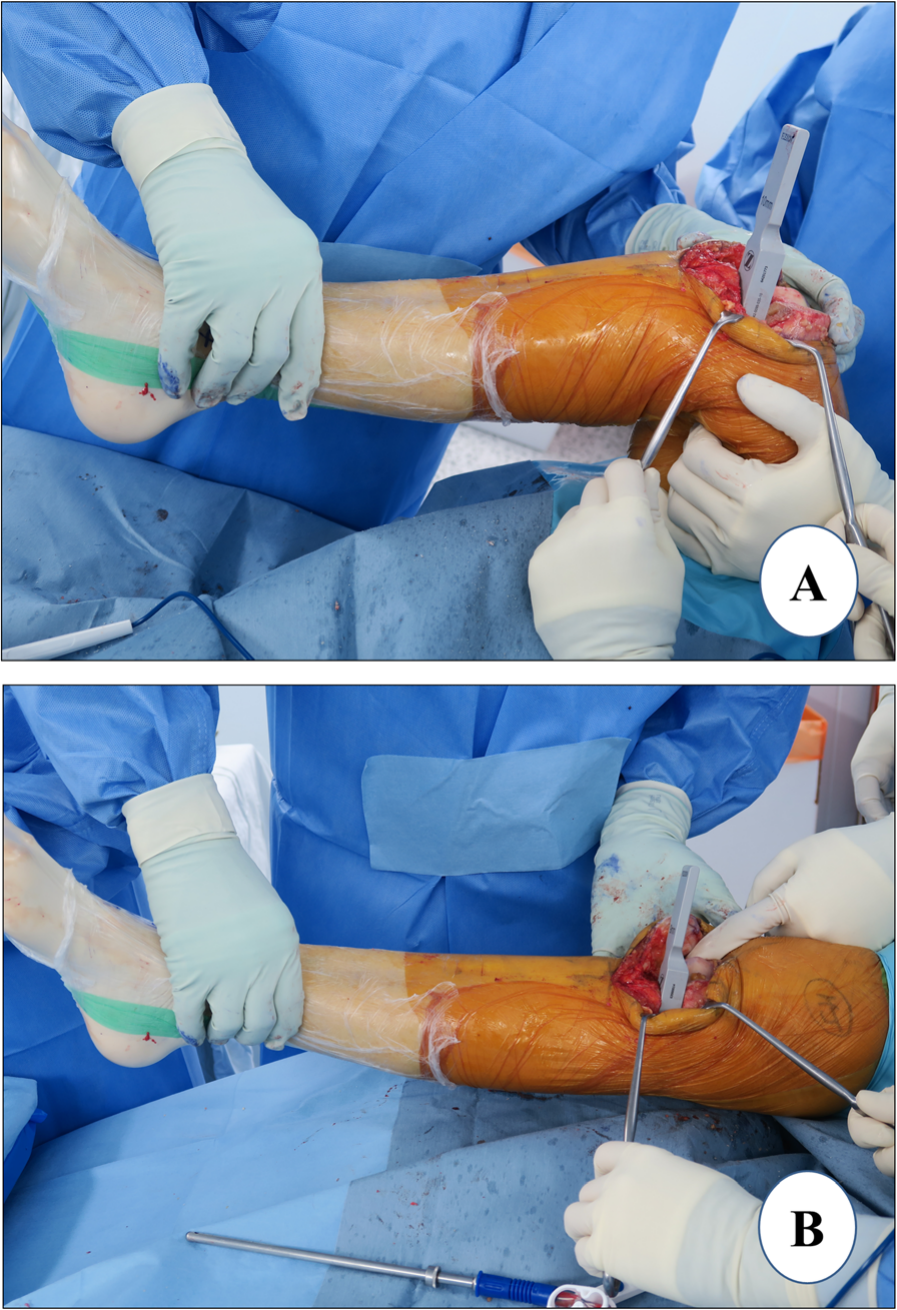
Gap assessment. **a** flexion gap check and **b** extension gap check

The residual meniscus is resected, osteophytes are removed, and the residual bone fragment of the medial tibial plateau is completely cut. The flexion (Fig. 5a) and the extension (Fig. 5b) gaps are confirmed to be equal using gap blocks which are usually 9 or 10 mm thick. If both flexion and extension gaps are too tight, a revision of the proximal tibia cut of 2 mm should be considered. *The PPK prosthesis system has a 2-mm cutting guide which could be applied over the headless trocar pins of the tibia to revise the tibial cut precisely by 2 mm in thickness*. If only the flexion gap is tight, a revision cut to increase the posterior slope of tibia is considered. In the authors’ experience, such mismatch of the flexion-extension gap rarely occurs provided that the previous steps are executed meticulously.

### Tibial sizing and final tibial preparation

**Fig. 6:**
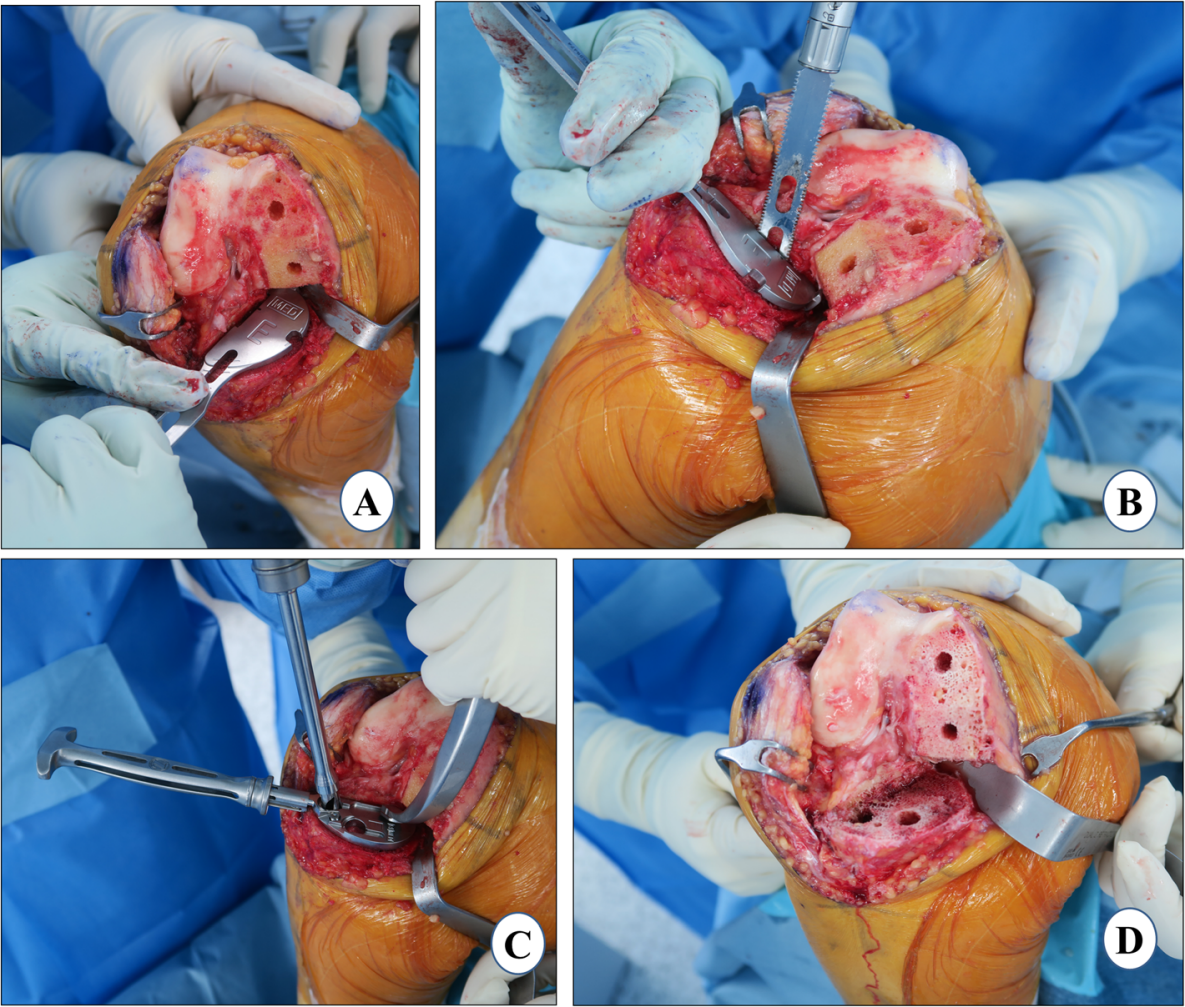
Tibial sizing and final tibial preparation. **a** tibial sizing, **b** tibial component keel cut, **c** tibial preparation, and **d** tibial and femoral surfaces after peg holes

An appropriate tibial sizer is inserted that best covers the resected proximal tibia in both the anteroposterior (AP) and the mediolateral dimensions (Fig. 6a). The tibial sizer should be placed flush to the medial cortex (Fig. 6b). If the sizer overhangs, confirm that the vertical tibial resection is as far lateral as possible; or use a smaller tibial size. The medial overhang may cause pain and should be avoided [65, 66]. If the tibial sizer has a medial overhang of 2 mm or more and the AP dimension is correct, an additional removal of the lateral part of the remaining tibia should be considered. Once the appropriate size tibial trial is decided, it is placed flush to the tibial surface and the drilling (Fig. 6c) of the holes for the tibial component pegs is performed (Fig. 6d).

#### Evidence

The ideal scenario for optimal tibial component fixation is to have the best fit in the AP and mediolateral dimensions without impingement, to prevent tibial-plateau fracture or future component loosening. Unfortunately, this is often not possible, partly because of mismatch between the prosthesis design and bone anatomy [67]. Inadequate bony support of the proximal tibia to the tibial component is associated with its early subsidence or loosening [68]. A cortical bony coverage of the tibial component in the posteromedial and anterolateral region is strongly recommended for optimal fixation [69]. In addition, surgeons must avoid the tibial component overhang of 3 mm or more, as this severely compromises the outcome [68].

### Trial test and polyethylene insert selection

**Fig. 7:**
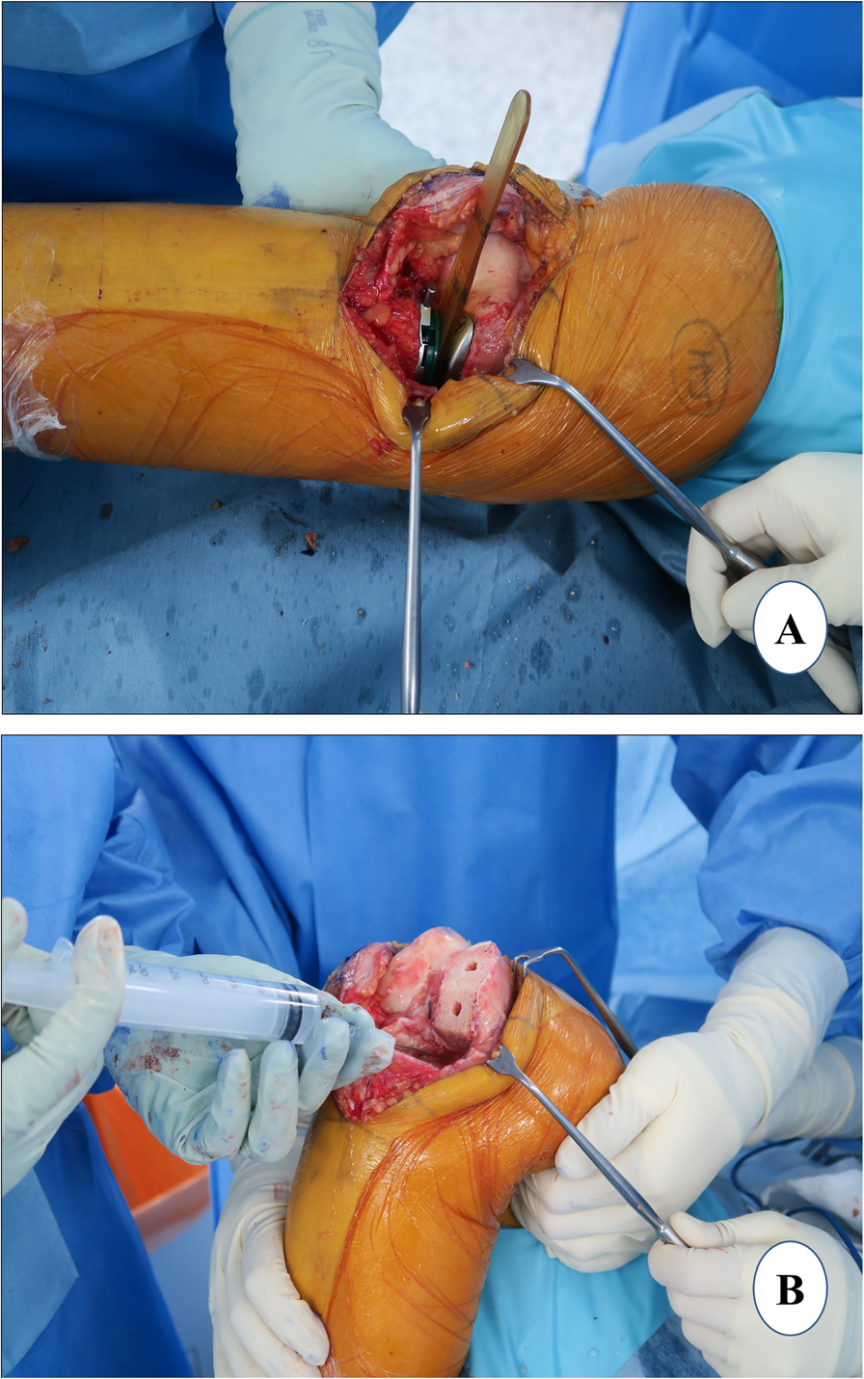
Trial test and polyethylene insert selection. **a** trial test with 2 mm gauge and **b** periarticular injection and trial test

When all the bone surfaces are prepared, a trial reduction is performed with the selected size of femoral, tibial, and polyethylene trials. The knee is manipulated through a full range of motion to determine the stability of the joint. A 2-mm end of the tension gauge/“amber stick” is inserted in flexion and extension to ensure that the gaps are not too tight (Fig. 7a). It is important to ensure a slight under-correction of the limb alignment and have appropriate ligamentous tension restored (2–3 mm of laxity) in flexion and extension. A 2-mm blade should be able to insert easily but snugly, whereas 3-mm blade insertion should be difficult. Before final implantation, the limb alignment is checked. Once the size and thickness of the components, and the limb alignment are confirmed, the trial components are removed. An irrigation of cut bone surfaces is done with 1 L of normal saline using a pulse lavage system to remove debris and fat. All cement-receiving bone surfaces are dried with a clean lap sponge. Of note, a 50-cc periarticular cocktail injection comprising 1 mg epinephrine, 30 mg ketorolac, 200 mg ropivacaine, 10 mg morphine, 250 mg cefuroxime, and the remaining normal saline is prepared just before surgery. A 25-cc volume of this periarticular injection cocktail is injected in the MCL and posterior capsule (Fig. 7b).

### Implant cementation

*A single 40-g unit of cement (Refobacin® Bone Cement R, Zimmer Biomet, Warsaw, IN, USA) is used for implantation of both the components. The tibial cementation is immediately followed by femoral cementation and both are performed in a single cement-curing time duration*.

#### Tibia cementation

**Fig. 8:**
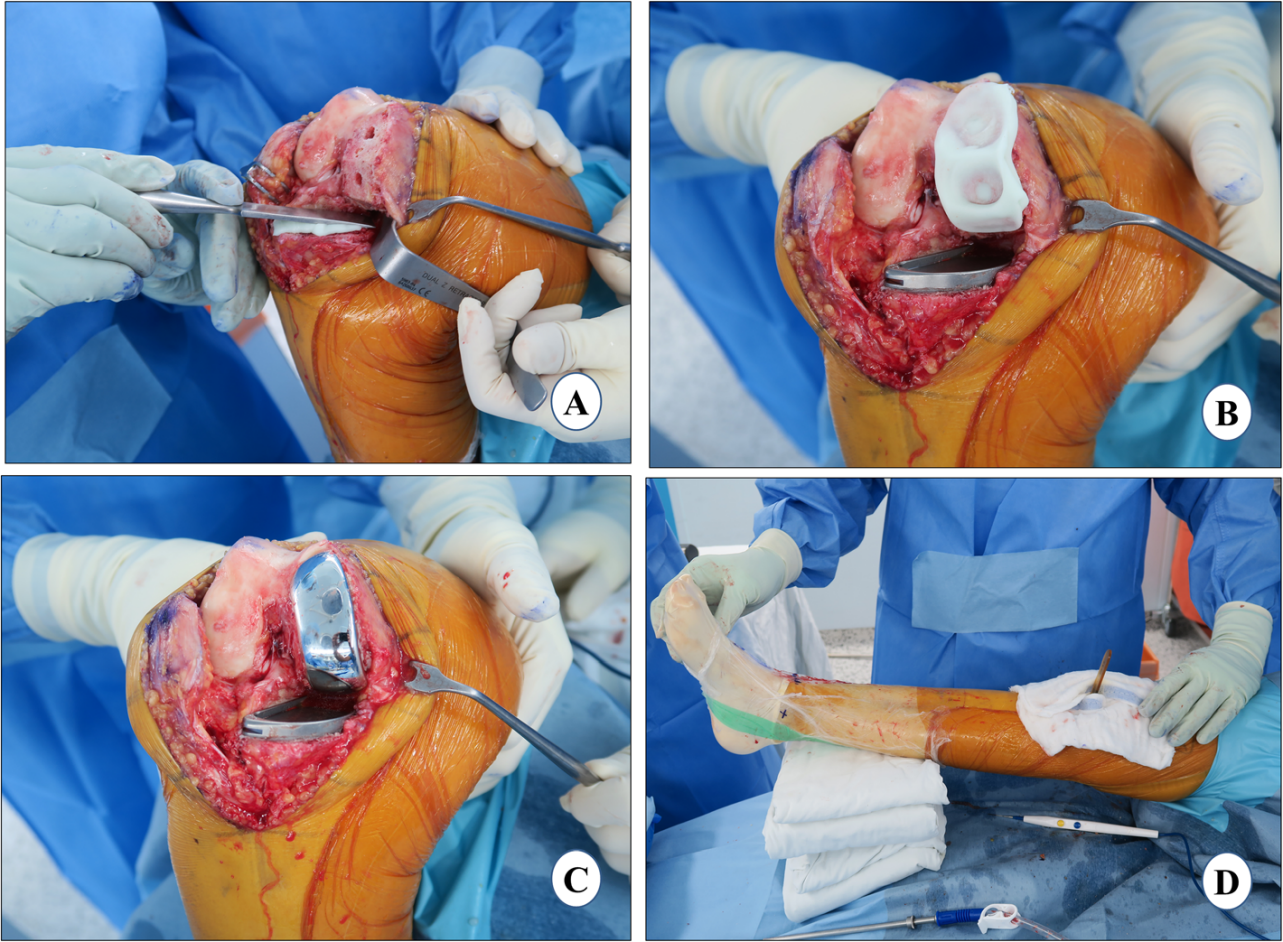
Implant cementation. **a** tibial cementation, **b** femoral cementation, **c** Implantation, and **d** waiting for cement curing

While in flexion, the tibia is levered out anteriorly with the help of a retractor posteriorly with external rotation to facilitate proper cementation and implantation. A single 40-g unit of cement is vacuum mixed. As soon as cement properties permit, a thin layer of cement is applied over the entire underside of the tibial component and lateral flange. The cement should just overfill the pockets on the underside of the tray, up to 1 mm proud posteriorly and 2 mm proud anteriorly. Contamination of the component-cement interface must be avoided. With the flat nozzle of the cement gun, the tibia is cemented and pressurized manually with a broad, flat osteotome from the posterior to the anterior direction, striving for penetration of 3–4 mm (Fig. 8a). While placing the final component, the posterior portion of the tibial component is gently pressed down followed by the anterior portion of the component using a rasp handle. This maneuver will force excess cement anteriorly and prevent the posterior extrusion of cement where it becomes difficult to remove. The tibial component should be gently pressed and not hammered, as it may lead to fracture of the tibial plateau. Any excess cement should be removed from the posterior and anterior aspects of the tibia using a curved curette. It should be confirmed that all the cement has been removed from the proximal surface of the tibial component especially posteriorly as this will prevent the proper assembly of the polyethylene component.

#### Femur cementation

The femoral surface is dried, and a layer of cement is applied to the distal femur with the flat nozzle of cement gun. The cement is manually spread in the peg holes and posterior chamfer facet and pressurized, striving for a penetration of 3–4 mm (Fig. 8b). A thin layer of cement is also applied over the underside of the femoral component. With the leg in deep flexion, the posterior (longer) peg of the femoral component is inserted first followed by the anterior one. The femoral component is impacted with a mallet. It must be ensured that the anterior peg is engaged, and the component is fully seated (Fig. 8c). The excess cement is removed especially from the posterior aspect with the help of curette and wet gauze. A 1-mm thinner trial than the final bearing is inserted with a 2-mm tension gauge to reduce excessive pressure on the cement which can result in cement leakage. It is then necessary to wait for the cement curing with the knee in extension (Fig. 8d). After the cement is cured, the trial insert is removed, and any remaining excess cement is curetted out before the final placement of the tibial bearing.

#### Evidence

Cement fixation allows the load to be distributed more evenly across the tibia, even with malalignment. Without cement fixation, there is an increased risk of point loading resulting in aseptic loosening [70]. *Of note, a systematic review of 10 studies has reported good clinical outcomes with cementless UKA* [71]*. Nonetheless, we routinely use cemented prosthesis for all our knee arthroplasties, and this is supported by long-term studies* [32, 72–74] *and over a decade of successful results in our patients*.

### Polyethylene insert and wound closure

The correct size and thickness of the final polyethylene bearing component is confirmed by checking the gap balance with the trial bearings in maximum flexion and extension. The tension gauge/“amber stick” is used to assess adequate laxity in flexion and extension. Then alignment is rechecked to verify that the joint has not been overcorrected. The knee is irrigated for the final time and the final tibial bearing is inserted with the knee in 60–70° of flexion.

**Fig. 9:**
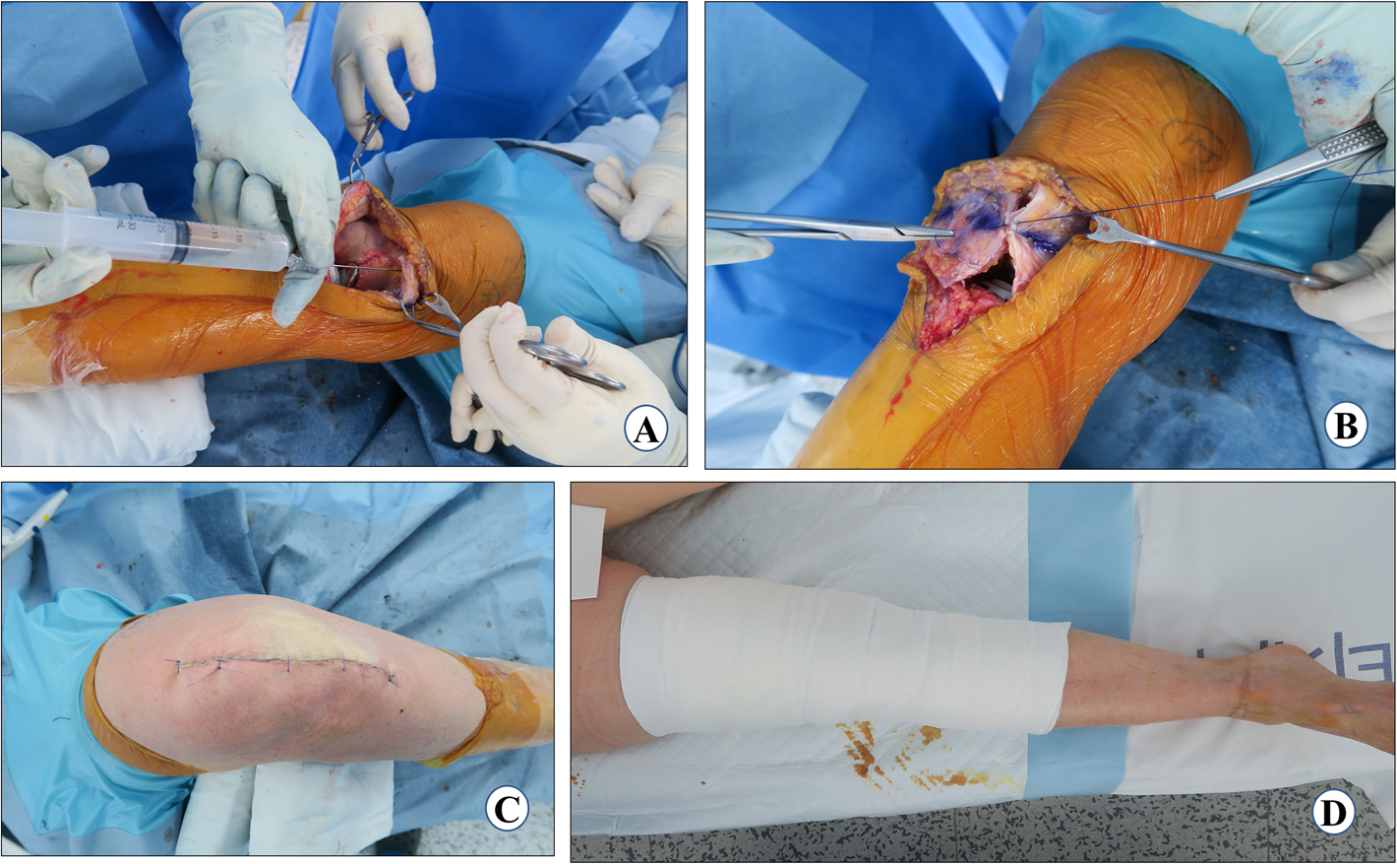
Polyethylene insert and wound closure. **a** periarticular injection, **b** capsule closure, **c** skin closure, and **d** compression bandage and post-operative limb alignment

Another 25 cc of cocktail injection is injected into the medial capsule and quadriceps muscle (Fig. 9a). An airtight wound closure should be the goal. The knee-joint capsule is closed with number 1 vicryl in approximately 30° of knee flexion (Fig. 9b). Thirty milliliters of 3 g tranexamic acid in 30 ml solution is injected into the knee joint [75] and then the knee is flexed to confirm the tight closure so that fluid is not leaking through any suture site. Next, subcutaneous sutures are applied meticulously with the help of 3–0 vicryl in 90° of flexion. The skin is closed with prolene 3–0 using a subcuticular continuous suture technique (Fig. 9c). If a tourniquet was used, it is deflated at this point and the wound is inspected for any blood oozing. *If not, then fibrin glue (Exofin, Chemence Medical, Alpharetta, GA, USA) is applied to seal the skin wound. An occlusive dressing material is applied followed by a compression bandage* (Fig. 9d).

In addition to good surgical technique, postoperative rehabilitation plays an important role in the recovery of the patient after UKA. Rehabilitation should ideally start before surgery. The authors’ preferred approach is to offer preoperative seminars and educational videos regarding the procedure, and future course [76]. Although controversial, preoperative rehabilitation can also play an important role in strengthening the quadriceps muscle of the patient and conditioning them for post-surgery physiotherapy. In the authors’ experience, preoperative rehabilitation also reduces the anxiety of the patient. In general, the rehabilitation guidelines for UKA are similar to what is followed for TKA. However, the surgeon should be cautious when the patient is severely osteoporotic or more thick bone is resected from the tibia than recommended (~ 4 mm). In these scenarios, rehabilitation should be less aggressive, and the knee should be protected with a brace and the use of crutches for ambulation. Most of the patients are able to return to routine daily activities by 2–3 months and return to sports by 6 months [77]. Rehabilitation should achieve both physical and mental well-being, with a healthy, long life as the ultimate goal.

## Conclusions

The adoption of a step-wise and meticulous approach is critical for successfully performing UKA surgery. Exposure, tibial cut, cementation, and wound closure are the most important aspects of the UKA surgical technique. The exposure should be optimal (not minimal) so that all the landmarks are visualized properly, and adequate space is available to prevent errors in bony cuts. In the spacer block method, the tibial cut plays a vital role in achieving preplanned limb alignment and component position (tibial as well as femoral). *A varus of 1–4° in the final mechanical hip-knee-ankle alignment is desirable for optimal results*. Even distribution of the cement is important for adequate fixation. An airtight layer-by-layer wound closure is necessary for prevention of wound problems and early rehabilitation.

## Data Availability

Not applicable
